# Different concentrations of lipopolysaccharide regulate barrier function through the PI3K/Akt signalling pathway in human pulmonary microvascular endothelial cells

**DOI:** 10.1038/s41598-018-28089-3

**Published:** 2018-07-02

**Authors:** Xia Zheng, Wang Zhang, Xiaotong Hu

**Affiliations:** 1 Department of Critical Care Medicine, The First Affiliated Hospital, College of Medicine, Zhejiang University, Hangzhou, Zhejiang 310003 P.R. China; 2 State Key Laboratory for Diagnosis and Treatment of Infectious Diseases, Collaborative Innovation Center for Diagnosis and Treatment of Infectious Diseases, The First Affiliated Hospital, College of Medicine, Zhejiang University, Hangzhou, Zhejiang 310003 P.R. China

## Abstract

Lipopolysaccharide (LPS) can lead to vascular endothelial barrier dysfunction, which often results in acute lung injury and acute respiratory distress syndrome. However, the effects of different concentrations of LPS on human pulmonary microvascular endothelial barrier function and the involvement of the phosphatidylinositol-3-kinase-serine/threonine kinase (PI3K/Akt) pathway in this process remain unclear. Human pulmonary microvascular endothelial cells (HPMECs) were stimulated with different doses of LPS, and barrier function was examined by determining cell monolayer permeability, cell migration, and the expression of intercellular junction proteins (VE-Cadherin, Claudin-5, and Connexin-43). LY294002 was used to inhibit PI3K to verify the role of the PI3K/Akt pathway in the regulation of barrier function in HPMECs stimulated by LPS. Low doses of LPS increased HPMEC migration, up-regulated VE-Cadherin and Claudin-5 expression, down-regulated Connexin-43 expression, and promoted Akt phosphorylation, which could collectively decrease monolayer permeability. In contrast, high doses of LPS suppressed HPMEC migration, down-regulated the expression of VE-Cadherin and Claudin-5, up-regulated Connexin-43 expression, and reduced Akt phosphorylation, which could collectively increase monolayer permeability. LPS has a biphasic effect on HPMEC barrier function through the PI3K/Akt pathway, and this effect is concentration-dependent.

## Introduction

Sepsis is a life-threatening condition resulting from organ dysfunction due to a dysregulated host response to infection^1^. It is one of the major causes of death in critically ill patients^2^. The lungs are the organs most vulnerable to sepsis, and the treatment of sepsis patients is often complicated by acute lung injury (ALI) or acute respiratory distress syndrome (ARDS), both of which have high mortality rates^3^. Lipopolysaccharide (LPS), the main component of the cell walls of Gram-negative bacteria, destroys the barrier function of human pulmonary microvascular endothelial cells (HPMECs) and plays an important role in the development of sepsis. Disruption of the pulmonary endothelial barrier is a hallmark of sepsis and ALI/ARDS. Dynamic damage to the microvascular barrier structure and function is involved in acute inflammatory responses, as well as in the isolation and clearance of pathogenic microorganisms^4^. Leakage of the pulmonary microvascular system leads to the exudation of large amounts of protein-rich oedema fluid, and vascular leakage caused by endothelial inflammation allows the entry of innate immune cells and humoral effector molecules. Yet severe exudation can also lead to sepsis-related ALI/ARDS in septic patients, which is characterized by intractable hypoxemia, respiratory distress and progressive respiratory failure^5^.

HPMECs are the cells that are the main target and effector involved in lung injury and this can also be induced by LPS *in vitro*. Barrier hyper-permeability as a model for LPS-challenged barrier dysfunction is the result of a complex interaction between endogenous pro-inflammatory cytokines, including tumour necrosis factor (TNF-α) and interleukin-1 (IL-1), and inflammatory cells, including circulating neutrophils and pulmonary-resident alveolar macrophages^6,7^. Endothelial barrier dysfunction occurs due to interrupted cell-cell junction formation, reduced cell migration and proliferation. The clinical severity and prognosis differ in ALI and ARDS patients. Therefore, to investigate the differences of clinical severity in ALI and ARDS, we examined the effect of different concentrations of LPS on HPMECs.

Previous studies have shown that the cell-cell junction formation, cell migration and cell proliferation changes associated with endothelial barrier dysfunction, are mediated by the PI3K/Akt pathway^8–10^. The PI3K/Akt pathway is the main regulator of endothelial barrier function in HPMECs^6^. Various drugs such as Omentin and Paeoniflorin may reduce barrier leakage during sepsis by inhibiting the PI3K pathway, and this pathway is a key target for therapeutic intervention^10,11^. The PI3K/Akt pathway also mediates endothelial cell migration through actin cytoskeleton reorganization^12^. In addition, reduced Akt phosphorylation can inhibit human umbilical vein endothelial cell proliferation and migration^13^. Recent studies showed that the progressive reduction of Akt phosphorylation may contribute to impaired endothelial function through different downstream effectors, and that this reduction could play a crucial role in the pathophysiology of sepsis^14,15^. In contrast, other studies have shown that increased Akt phosphorylation induced by LPS can result in hyper-permeability in endothelial cells^11^. However, the effects of different LPS concentrations on the activation of PI3K/Akt in HPMECs have not been determined. In addition, the relationship among the PI3K/Akt pathway, cell migration, and the expression of intercellular junctions in HPMECs has not been fully elucidated. Therefore, this study investigated whether HPMECs treated with different concentrations of LPS displayed different levels of barrier dysfunction induced by signal cascade activation.

## Results

### HPMEC monolayer permeability after treatment with different concentrations of LPS

**Figure 1:**
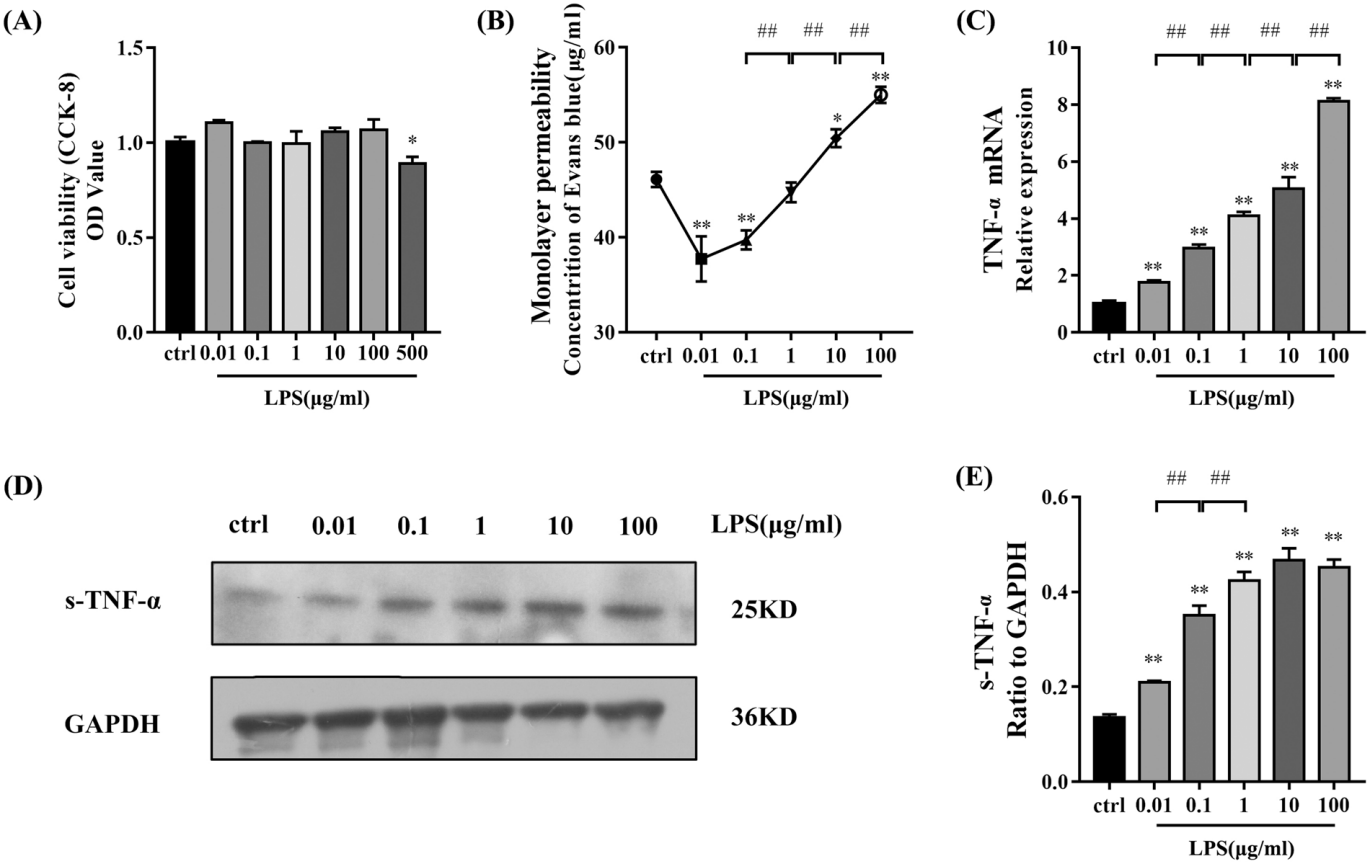
Changes in cell proliferation, monolayer permeability, and tumour necrosis factor-α (TNF-α) in HPMECs treated with different concentrations of LPS. (**A**) Proliferation was measured using a CCK-8 kit. There was no significant difference between each group except for the 500 μg/ml LPS group (P < 0.05). (**B**) Monolayer permeability was measured using a Transwell-Evans Blue (EB) assay. EB concentrations in the lower chambers were decreased in groups treated with 0.01 μg/ml (P < 0.01) and 0.1 μg/ml LPS (P < 0.01) and higher in groups treated with 10 μg/ml (P < 0.05) and 100 μg/ml (P < 0.01) LPS than in the control group. (**C**) Transcript levels of TNF-α in HPMECs treated with different concentrations of LPS. mRNA expression of TNF-α was upregulated with increasing LPS concentration compared with that in the control group (P < 0.01). (**D**,**E**) TNF-α protein concentration in the supernatant increased with increased LPS concentration compared with that in the control group (P < 0.01). Error bars represent SD (standard deviation), *P < 0.05 vs. control group, **P < 0.01 vs. control group, ^#^P < 0.05, ^##^P < 0.01.

The effects of different concentrations of LPS on HPMEC monolayer permeability were estimated using a Transwell-Evans Blue (EB) monolayer permeability assay. In addition, a CCK-8 assay was used to detect cytotoxicity of LPS in different concentrations (Fig. 1A). The results showed that, after 24 hours of treatment, EB concentrations in the lower chambers (representing monolayer permeability) were significantly lower in cells treated with 0.01 and 0.1 μg/ml LPS than in the control group. In contrast, EB concentrations in the lower chambers were significantly higher in the 10 and 100 μg/ml LPS groups than in the control group. There was no significant difference between EB concentrations in the 1 μg/ml LPS group and the control group (Fig. 1B).

### TNF-α levels in HPMECs after LPS treatment

To evaluate the effect of different LPS concentrations on the inflammatory response of HPMECs, mRNA and protein levels of TNF-α were determined by qPCR and Western blotting. The results showed that increasing LPS concentrations resulted in increased levels of TNF-α mRNA and protein compared with those in the control group (Fig. 1C–E). This suggests that the severity of the inflammatory reaction is related to LPS concentration.

### Migration of HPMECs treated with different concentrations of LPS

**Figure 2:**
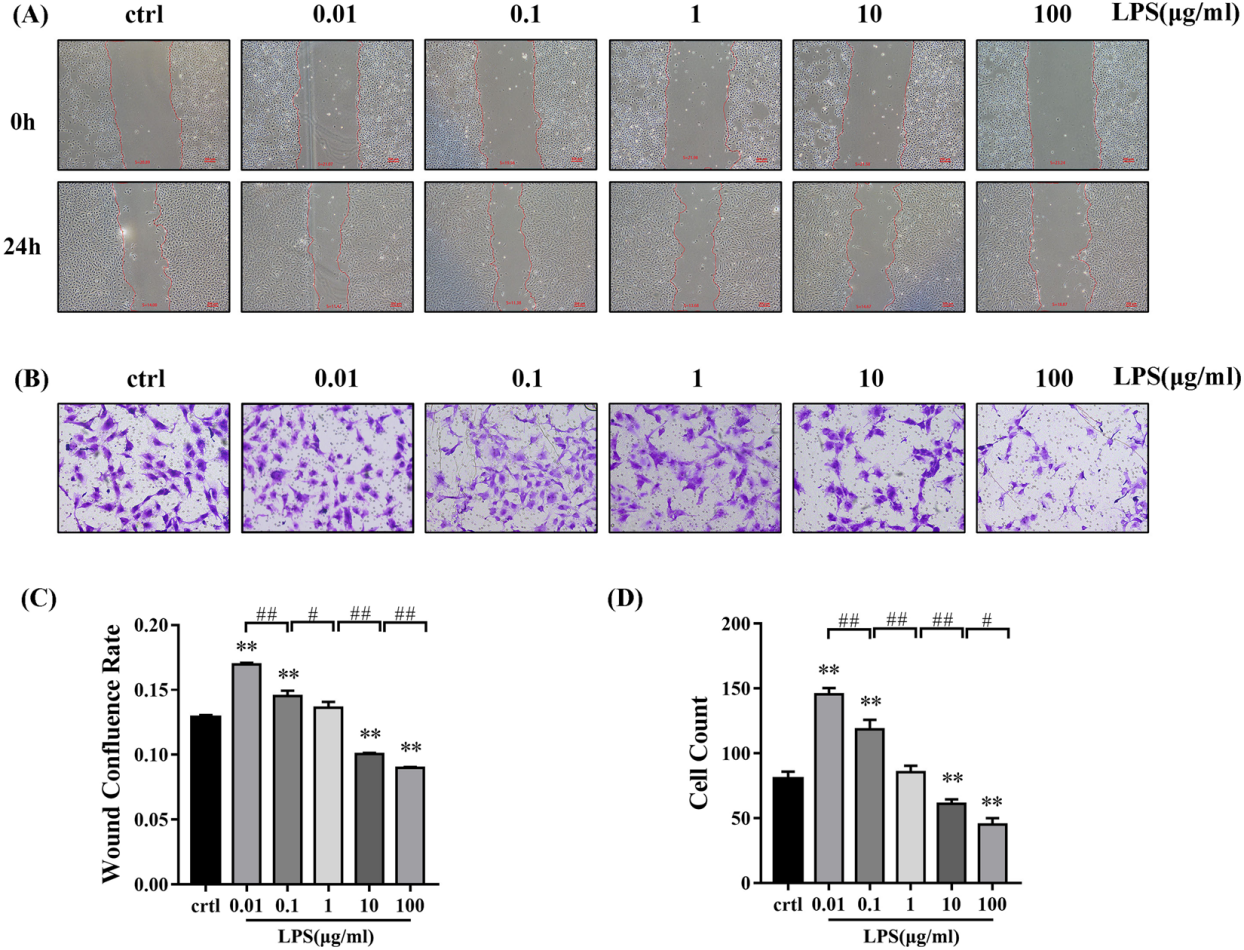
Migration of HPMECs treated with different concentrations of LPS. (**A**,**C**) The effects of LPS on scratch wound confluence in cultured HPMECs. (**A**) Representative images (**C**) Quantification of confluence rate at 24 h [% wound confluence = (a − b) × 100%/a; a = Initial scratch wound area at 0 h, b = Scratch wound area at 24 h]. Compared with that in the control group, cell confluence was significantly increased in the 0.01 (P < 0.01) and 0.1 μg/ml LPS (P < 0.01) groups and significantly decreased in the 10 (P < 0.01) and 100 μg/ml LPS (P < 0.01) groups. (**B**,**D**) A Transwell assay was used to measure migration. (**B**) Representative images. (**D**) Quantification of cell migration after 24 h. Cell numbers in the 0.01 (P < 0.01) and 0.1 μg/ml LPS groups (P < 0.01) were significantly higher and those in the 10 μg/ml (P < 0.01) and 100 μg/ml (P < 0.01) LPS groups were lower than in the control group.

To determine the effects of different concentrations of LPS on HPMEC migration, cell migration rates were measured using an *in vitro* wound healing assay (Fig. 2A,C). The results showed that after treatment with 0.01 or 0.1 μg/ml LPS for 24 h, the rates of scratch wound confluence were significantly increased compared with those in the control group. In contrast, the rates of scratch wound confluence were significantly decreased in the 10 and 100 μg/ml LPS groups. These results suggest that 0.01 and 0.1 μg/ml LPS induced HPMEC migration. Next, we determined the number of migrated cells in different groups using a Transwell migration assay (Fig. 2B,D). The results showed that the numbers of migrated cells in the 0.01 and 0.1 μg/ml LPS groups were significantly higher than in the control group, while there were a lower number of migrated cells in the 10 and 100 μg/ml LPS groups. There were no significant differences between the 1 μg/ml LPS group and the control group.

### Expression of intercellular junction proteins in HPMECs treated with different concentrations of LPS

**Figure 3:**
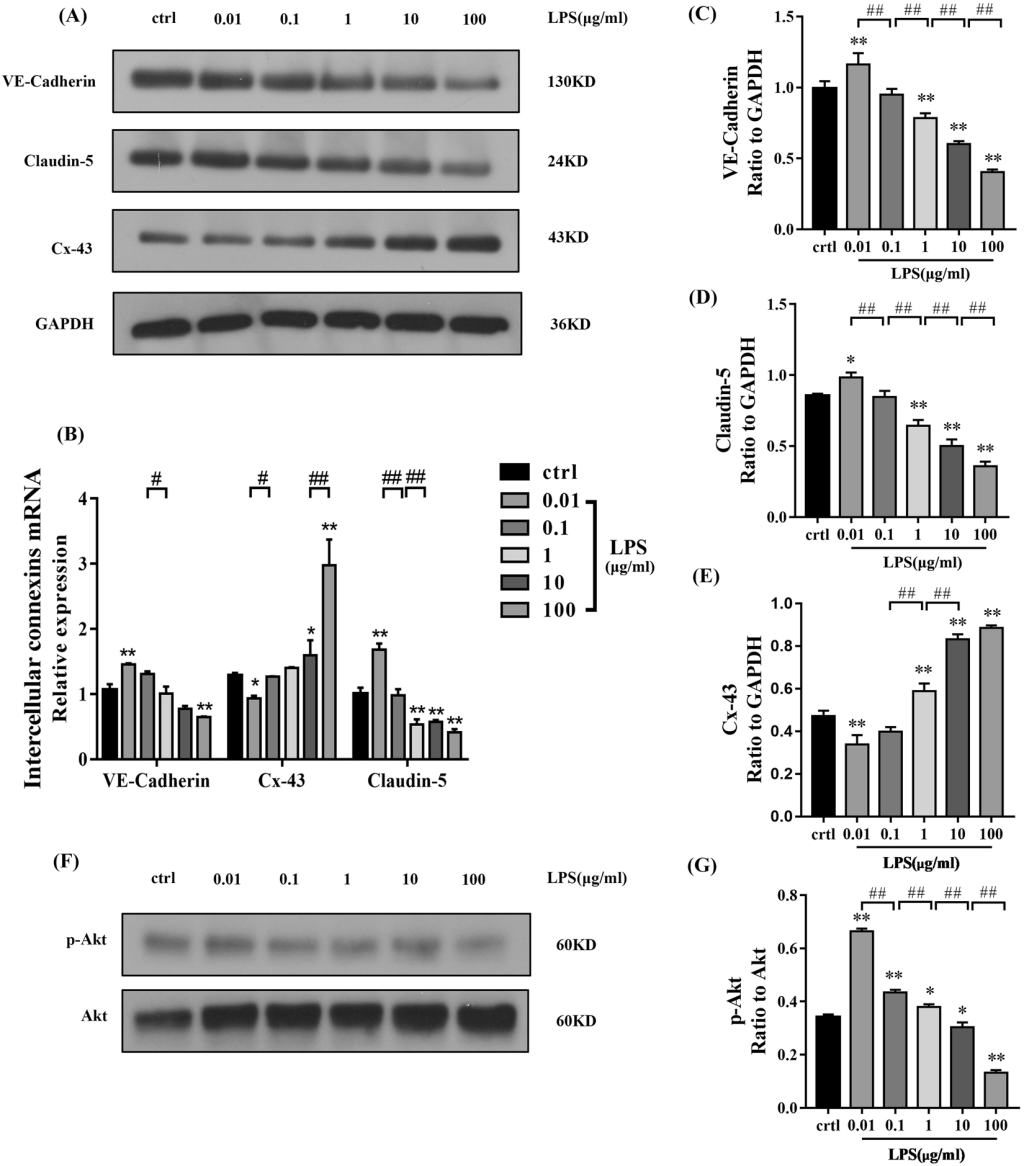
The effects of LPS on the expression of intercellular junctions and Akt phosphorylation in HPMECs. (**A**) Expression of intercellular junction proteins (VE-Cadherin, Cx-43, and Claudin-5) was evaluated by western blot. Relative protein levels are expressed as the ratio of the target protein to GAPDH. (**C**,**D**) The levels of VE-Cadherin and Claudin-5 were remarkably up-regulated after treatment with 0.01 μg/ml LPS (C: P < 0.01, D: P < 0.05) and down-regulated after treatment with 1 (P < 0.01), 10 (P < 0.01), and 100 μg/ml LPS (P < 0.01) compared with those in the control group. (**E**) Cx-43 levels were decreased after 0.01 µg/ml LPS treatment (P < 0.01) and up-regulated after 1 (P < 0.01), 10 (P < 0.01), and 100 μg/ml LPS (P < 0.01) treatment compared with those in the control. (**B**) VE-Cadherin and Claudin-5 mRNA transcript levels were up-regulated after 0.01 μg/ml LPS treatment (P < 0.01), but down-regulated after 100 μg/ml LPS (P < 0.01) treatment compared with those in the control group. Claudin-5 mRNA levels were also decreased after 1 (P < 0.01) and 10 μg/ml (P < 0.01) LPS treatment. Cx-43 mRNA levels were decreased in the 0.01 μg/ml LPS group (P < 0.05), but were increased in the 10 (P < 0.05) and 100 μg/ml LPS groups (P < 0.01) compared with those in the control. (**F**,**G**) The ratio of p-Akt to total Akt protein was increased in the 0.01 (P < 0.01), 0.1 (P < 0.01), and 1 μg/ml (P < 0.05) LPS groups, and was decreased in the 10 (P < 0.05) and 100 μg/ml (P < 0.01) LPS groups compared with that in the control group.

To determine the changes in HPMEC intercellular junctions after LPS treatment, the protein levels of VE-Cadherin, Claudin-5, and Connexin-43 (Cx-43) were measured. Western blotting showed that the protein levels of VE-Cadherin and Claudin-5 were remarkably up-regulated after treatment with 0.01 μg/ml LPS compared with those in the control group. In contrast, both Claudin-5 and VE-Cadherin were down-regulated after treatment with 1, 10, and 100 μg/ml LPS. The expression of Cx-43 showed the opposite trend after LPS treatment. There were no significant differences in the expression of VE-Cadherin, Claudin-5, or Cx-43 between the 0.1 μg/ml LPS group and the control group (Fig. 3A, C–E).

**Figure 4:**
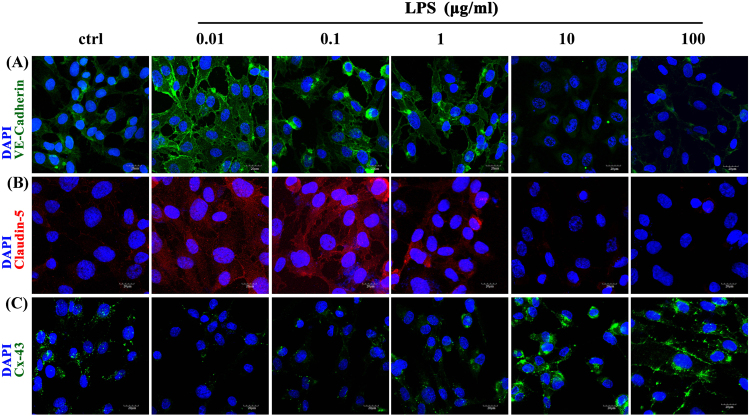
Immunofluorescence staining of VE-Cadherin, Claudin-5, and Cx-43 in HPMECs treated with 0.01, 0.1, 1, 10 and 100 μg/ml LPS. (**A**) The expression of VE-Cadherin after LPS treatment. Green represents VE-Cadherin and blue represents the nuclei. (**B**) The expression of Claudin-5 after treatment with different concentrations of LPS. Red indicates Claudin-5 and blue indicates nuclei. (**C**) The expression of Cx-43 after treatment with different concentrations of LPS. Green indicates Cx-43 and blue indicates nuclei. Scale bar: 20 μm

Similarly, mRNA levels (Fig. 3B) and fluorescence intensity (Fig. 4) of VE-Cadherin and Claudin-5 were increased after 0.01 μg/ml LPS treatment, however, decreased after 10 and 100 μg/ml LPS treatment compared with those in the control. Cx-43 mRNA levels and fluorescence activity showed the opposite results compared with Claudin-5 and VE-Cadherin.

### LPS-induced barrier function changes in HPMECs are dependent upon the PI3K/Akt pathway

To clarify whether the PI3K/Akt pathway is involved in the regulation of HPMEC barrier function after LPS treatment, changes in the PI3K/Akt pathway after LPS treatment were investigated. First, we measured the ratio of phosphorylated Akt (p-Akt) to total Akt protein to evaluate the degree of PI3K/Akt pathway activation (Fig. 3F,G). The ratio of p-Akt to total Akt in HPMECs was significantly higher in cells treated with 0.01, 0.1, or 1 μg/ml LPS compared with that in the control group. In contrast, the ratio was significantly lower in cells treated with 10 or 100 μg/ml LPS.

**Figure 5:**
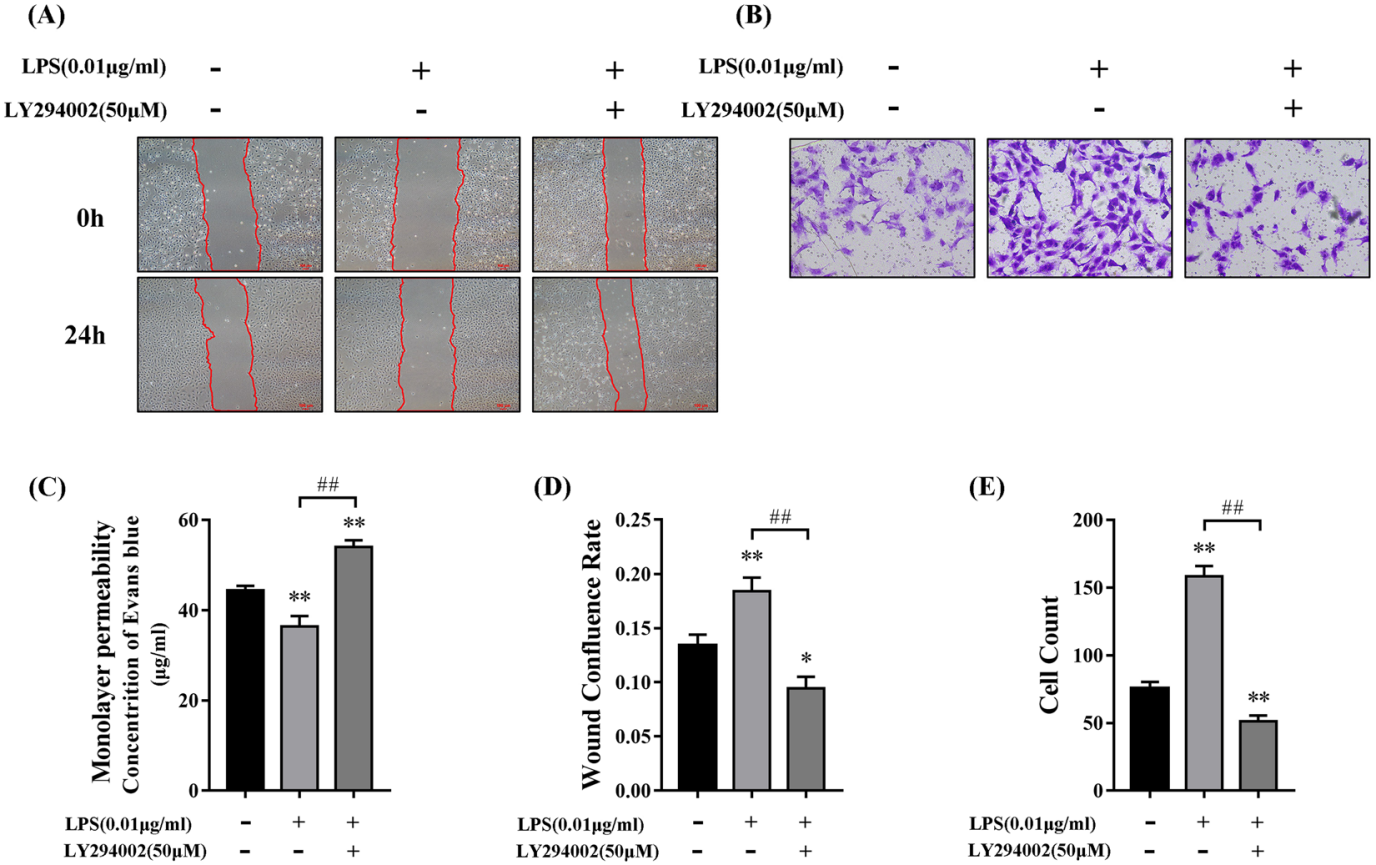
Monolayer permeability and migration of HPMECs after PI3K/Akt inhibition. (**A,D**) Wound healing assay (**A**) Representative images (**D**) Relative migration levels are presented as the scratch wound confluence rate after 24 h. The change of confluence rate in the LPS (0.01 μg/ml) group was significantly higher than in the control (P < 0.01) and LPS + LY294002 groups (P < 0.01). (**B,E**) Cell Transwell assay (**B**) Representative images (**E**) The number of migrated cells in the LPS (0.01 μg/ml) group was significantly higher than in the control (P < 0.01) and LPS + LY294002 groups (P < 0.01). (**C**) Monolayer permeability was measured by Transwell-EB assay; EB concentration in the LPS (0.01 μg/ml) group was lower than in the control (P < 0.01) and LPS + LY294002 groups (P < 0.01). There were no significant differences between the control group and LY294002 group (P > 0.05) in monolayer permeability, wound confluence rate and cell count (see Supplementary Fig. 1).

Next, we used LY294002 (50 μM), an inhibitor of PI3K/Akt signalling, to evaluate the effects of PI3K/Akt signalling on barrier function after LPS treatment. We chose to use LPS at a concentration of 0.01 μg/ml since this concentration resulted in the highest level of HPMEC migration and the largest p-Akt to Akt ratio. Monolayer permeability (Fig. 5C) in the LPS group was lower than in both the control and the LPS + LY294002 groups. In addition, the rates of scratch wound confluence in the LPS group were significantly higher than in the control and LPS + LY294002 groups (Fig. 5A,D). Similarly, the Transwell migration assay (Fig. 5B,E) revealed more migration in the LPS group than in the control and LPS + LY294002 groups. Together, these results suggest that LY294002 can counter the decreased permeability and increased migration induced by low doses of LPS treatment by inhibiting Akt phosphorylation.

**Figure 6:**
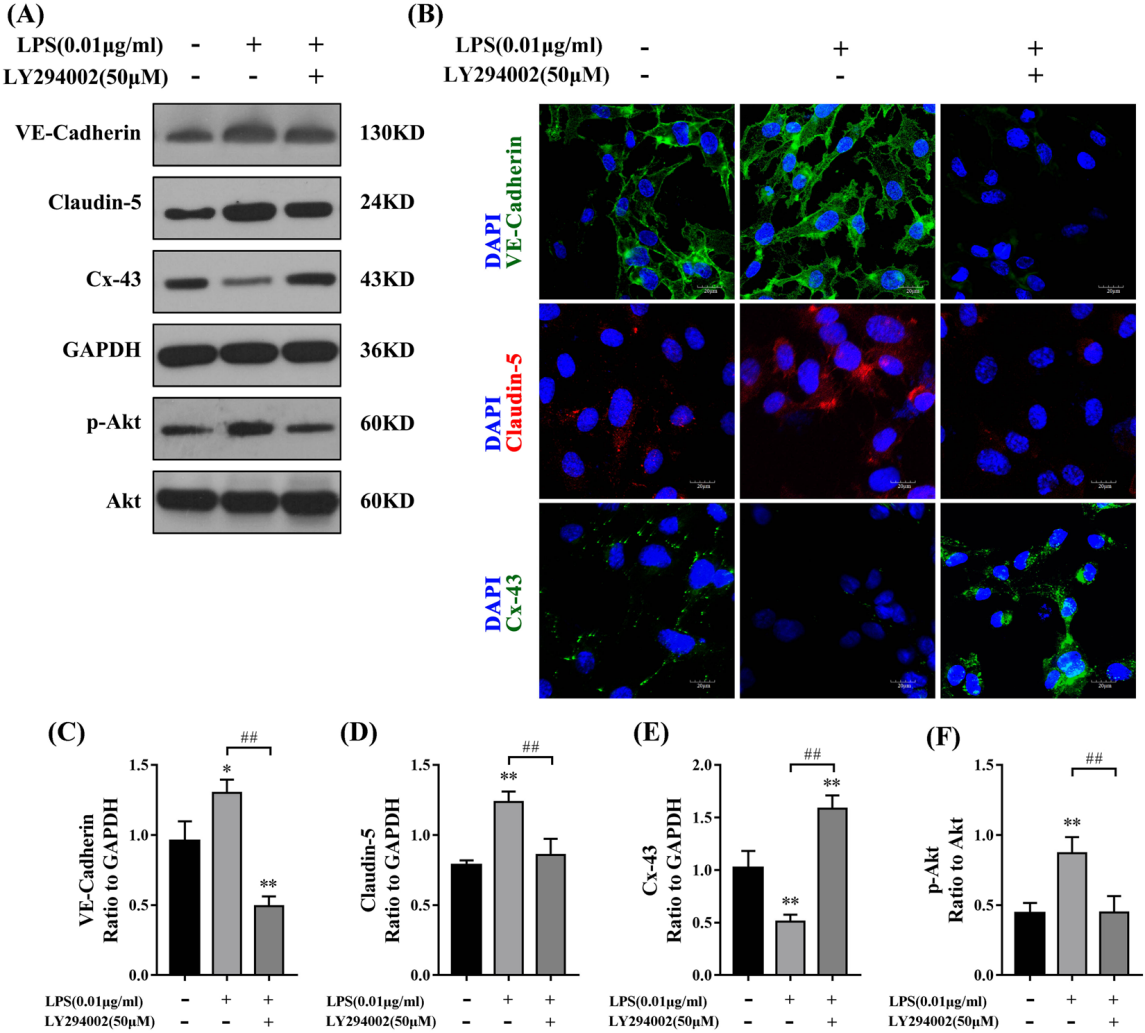
The expression of intercellular junction proteins after PI3K/Akt inhibition. (**A**) The expression of VE-Cadherin, Claudin-5, and Cx-43 was measured by Western blot analysis. (**C**,**D**) Relative protein levels are shown as the ratio of the target protein to GAPDH. The expression of VE-Cadherin and Claudin-5 in the LPS (0.01 μg/ml) group was increased compared with that in the control (C: P < 0.05, D: P < 0.01) and LPS + LY294002 groups (P < 0.01). (**E**) The expression of Cx-43 in the LPS (0.01 μg/ml) group was decreased compared with that in the control (P < 0.01) and LPS + LY294002 groups (P < 0.01). (**B**) Immunofluorescence staining of VE-Cadherin, Claudin-5, and Cx-43 in HPMECs treated with 0.01 μg/ml LPS and 50 μM LY294002. Green indicates VE-Cadherin and Cx-43, while red indicates Claudin-5 and blue indicates nuclei. (**A**,**F**) The ratio of p-Akt to total Akt in the LPS (0.01 μg/ml) group was higher than in the control (P < 0.01) and LPS + LY294002 groups (P < 0.01). Scale bar: 20 μm.

Lastly, we examined the expression of intercellular proteins involved in the PI3K/Akt pathway. The protein levels of VE-Cadherin and Claudin-5 in the LPS group were significantly higher than in the control and LPS + LY294002 groups (Fig. 6A,C,D), while Cx-43 showed the opposite results (Fig. 6A,E). p-Akt protein levels were also inhibited by LY294002 treatment (Fig. 6A,F). The fluorescence intensity of VE-Cadherin, Claudin-5, and Cx-43 reflected similar trends to the protein levels (Fig. 6B).

## Discussion

Endotoxin-induced sepsis involves extensive inflammation-induced endothelial dysfunction that can lead to the development of severe inflammatory disease. In general, endothelial dysfunction can be characterized as an alteration of cell migration and permeability. A previous study reported that LPS, an endotoxin, induced endothelial cell migration through the TLR-4/NF-κB pathway in a dose-dependent manner^16^. In a study on atherosclerosis, a chronic inflammatory disease of vessels, endothelial cell migration was shown to be involved in neovascularization and caused the plaques to become vulnerable to rupture^17^. LPS, which is typically used in ALI/ARDS models, damages and disrupts barrier function in activated endothelial cells. This can lead to the release of a variety of inflammatory mediators such as IL-1 and TNF-α, which are associated with increased migration and permeability of endothelial cells in sepsis and may be related to the increase in mononuclear macrophage transmembrane migration^18,19^. In our study, the results showed that lower doses of LPS increase cell migration, which may play a role in the ability of the endothelial barrier to repair itself. In contrast, higher doses of LPS reduced cell migration, and induced tissue injury and endothelial dysfunction, which can eventually lead to vascular leakage. The results are similar to those in a previous study that showed that stimulating PMVECs with lower doses of LPS increased proliferation, while higher doses of LPS led to the loss of cell viability^20^. Therefore, proper adjustment of HPMEC migration may be a therapeutic target for ALI/ARDS.

Enhancing cell migration and strengthening cell-cell connections is important for the recovery of barrier function. Cell-cell connections affect the adhesion and communication between adjacent endothelial and epithelial cells. Junctional complexes include tight junctions (TJs), adhesion junctions (AJs), and gap junctions (GJs). The main intercellular proteins in the endothelium include VE-Cadherin, Claudin-5, and Cx-43, and the expression of these proteins is closely related to vascular permeability^21^. VE-cadherin is the major transmembrane protein of endothelial cells, and plays a key role in maintaining and regulating endothelial permeability^22^. Previous studies of lung tissue specimens in clinical ARDS patients and cell models showed that reduced expression of VE-cadherin may be a major mechanism of increased vessel permeability in ARDS^23^. It was also reported that 10 μg/mL LPS stimulation was negatively correlated with VE-Cadherin at the endothelial cell membrane and positively correlated with monolayer cell permeability^24^. This is similar to our results, which showed up-regulation of VE-Cadherin and decreased membrane permeability at LPS concentrations of less than 1 μg/ml. Other studies showed that membrane permeability may be related to endocytosis and phosphorylation of VE-Cadherin^25–28^. Using the same model, we found that Cx-43 behaved oppositely to VE-Cadherin, with low LPS doses inducing decreased expression. Earlier studies showed that increased lung vascular permeability induced by inflammatory conditions may be amplified by increased levels of Cx-43 and intercellular communication among pulmonary endothelial cells^29^. Our research may complement other studies showing that LPS up-regulates Cx-43, thus inducing hyper-permeability of the cell monolayer^29–31^. One recent study showed that Poly (I:C) down-regulated Claudin-5 mRNA through the TLR3/TRIF pathway and increased endothelial monolayer permeability. In addition, the expression of Claudin-5 decreased in a dose- and time-dependent manner, indicating that Poly (I:C) induces dysfunction of the human pulmonary endothelial barrier by disrupting tight junctions^32^. We obtained similar results and showed that a low concentration of LPS up-regulated Claudin-5 mRNA and protein expression. In contrast to the known harmful effects of LPS, we found that different levels of LPS had dynamic effects, which may reflect disease severity. Interestingly, low levels of LPS seemed to induce barrier healing, while high levels of it induced barrier damage and inhibited healing.

We used the PI3K inhibitor LY294002 to determine whether the PI3K/Akt pathway is involved in the LPS-induced barrier function changes. Recent research has shown that activation of the PI3K/Akt pathway is associated with the suppression of inflammation, apoptosis, and reactive oxygen species (ROS) production in pulmonary endothelial cells, which could reverse barrier dysfunction^33^. Some studies have shown that inhibition of the PI3K/Akt pathway plays a key role in the amelioration of ALI/ARDS^10,33–35^. In this study, we found that LPS induced PI3K/Akt pathway inhibition or activation, which subsequently led to changes in HPMEC barrier function. Therefore, we hypothesized that low doses of LPS would lead to an addition in cell migration and increased cell-cell junction formation. We inhibited PI3K in HPMECs using LY294002 and demonstrated that the permeability of the LY294002 + LPS group was significantly higher than that of the LPS group. This indicates that PI3K/Akt pathway inhibition reduced the repair ability induced by low doses of LPS. Next, it was important to determine the effect of PI3K inhibition on monolayer permeability, cell migration, and intercellular junction protein expression in HPMECs. Similar to previous results, cell migration was reduced in the LY294002 + LPS group compared with that upon LPS treatment alone. In addition, some previous studies reported that the expression of intercellular junction proteins is regulated by the PI3K/Akt pathway^9,36^. Specifically, previous studies showed that VE-Cadherin inhibited the Claudin-5 transcriptional repressor FoxO1 via the PI3K/Akt pathway, thus upregulating Claudin-5 expression^37^. Similar results were observed in our study, and we found that PI3K/Akt inhibition downregulated Claudin-5 and VE-Cadherin expression while upregulating Cx-43 expression. Therefore, we believe that the increased migration and intercellular junction changes induced by PI3K/Akt activation is a mechanism of post-injury repair or self-healing in HPMECs. Other previous studies reported that LPS-induced differences in PI3K/Akt activation are related to membrane cell receptors such as Toll-like receptor 4 (TLR4) and platelet-derived growth factor receptor (PDGFR) in endothelial cells^38–40^. However, the specific mechanisms require further research. One limitation of our study is that we did not detect the self-healing or injury effects of LPS on barrier function *in vivo*, so this should be addressed in further studies.

In conclusion, our results suggest that LPS acts through the PI3K/Akt pathway to affect endothelial barrier function in a concentration-dependent manner. Low doses of LPS induce a potent recovery capacity while high doses of it induce injury in HPMECs. The migration of HPMECs and the expression of intercellular junction proteins are regulated by the activation of PI3K/Akt and can lead to changes in vascular endothelial cell permeability and barrier function.

## Methods

### Cell culture

HPMECs (Cat. No. 3000; ScienCell, San Diego, CA, USA) in Endothelial Cell Medium (ECM, Cat. No. 1001; ScienCell) containing 5% foetal bovine serum (FBS, Cat. No. 0025; ScienCell), 1% endothelial cell-derived growth factor (ECGS, Cat. No. 1052; ScienCell), and 1% penicillin/streptomycin (P/S, Cat. No. 0503; ScienCell) were incubated at 37 °C and 5% CO_2_. LPS (LPS from Escherichia coli 055: B5, Cat. No. L2880; Sigma-Aldrich, Steinheim am Albuch, Germany) and PI3K inhibitor LY294002 (#9901; Cell Signaling Technology, Danvers, MA, USA) were diluted with ECM without FBS. LPS was added to HPMECs for 24 hours at a concentration of 0.01, 0.1, 1, 10, or 100 μg/ml. Cells were treated for 24 hours with LY294002 at a concentration of 50 μM.

### Cell viability assay

HPMECs were grown in 96-well plates at a density of 5000 cells/well. Once the cells had adhered, LPS or LY294002 was added to each well. A Cell Counting Kit-8 (CCK-8; Dojindo, Tokyo, Japan) solution (10 μl) was then added to each well and incubated for 2 hours. Absorbance was then determined at 450 nm using a micro-plate reader (Spectra Maxi3x; Molecular Device, CA, USA). We repeated the experiments three times for each group and averaged the results.

### Wound healing assay

HPMECs were seeded in six-well plates as previously described. The cell monolayer was scratched using a 200 μl pipette tip before washing three times with phosphate-buffered saline (PBS) to clear cell debris and floating cells. One thousand microliters of serum-free ECM with different concentrations of LPS or LY294002 was then added, and the cells were incubated for 24 h at 37 °C in 5% CO_2_. Images were captured under a microscope before and after the 24 h incubation at the same position. Migration ability was measured by calculating the rate of scratch wound confluence after 24 h using Adobe Photoshop 2016 software (Adobe Systems Inc., San Jose, CA, USA).

### Transwell migration assay

Transwell inserts (#3464, pore size: 8.0 μm, CoStar; Corning Inc., Corning, NY, USA) were used for the migration assay. HPMECs were seeded in Transwell inserts at a concentration of 1 × 10^4^ cells/well in 100 μl of serum-free ECM. Different concentrations of LPS or LY294002 were then added to the upper chamber and 500 μl of complete ECM was added to the lower chamber. The plates were incubated for 24 h at 37 °C in 5% CO_2_. The cells were then washed three times with PBS and the Transwell inserts were fixed in formalin for 20 min. A cotton swab was then used to remove the non-migrating cells and the surface of the insert was washed three times with PBS. Next, the migrated cells on the lower surface of the inserts were stained with crystal violet for 10 min and rinsed three times with PBS. The cells were observed under a microscope and three random fields (×100) were chosen for each sample. The cell number was quantified and the average number of migrated cells from the three fields was compared between groups.

### Transwell-Evans Blue monolayer permeability assay

Transwell inserts (#3472, pore size: 3.0 μm, CoStar; Corning Inc.) were used for the permeability assay. HPMECs were seeded in Transwell inserts at a concentration of 1 × 10^4^ cells/well in 100 μl of complete ECM and incubated for 48 h at 37 °C in 5% CO_2_. 100 μl of serum-free ECM containing different concentrations of LPS or LY294002 was then added, and the cells were incubated for 24 h at 37 °C in 5% CO_2_. EB (Cat. No. E2129; Sigma)-conjugated albumin (final concentration: 0.67 mg/ml) was prepared by diluting a stock solution of 2% EB in a 60-fold excess of bovine serum albumin (BSA, 4%) to eliminate any free EB, as previously described^41^. 100 μl of EB-conjugated albumin was then added to the upper chamber, and 500 μl of 4% BSA was added to the lower chamber. The height of the EB-conjugated albumin in the upper chamber and the height of the 4% BSA in the lower chamber were kept the same to eliminate the influence of a hydrostatic pressure gradient. After incubating for 1 h at 37 °C in 5% CO_2_, the liquid in the lower chamber was collected. Finally, absorbance was determined at 620 nm using a micro-plate reader. We repeated the experiment in three separate wells and calculated the average. A standard curve was drawn according to the absorbance and the calculated leakage of total EB-albumin from the upper compartment to the lower compartment.

### Quantitative real-time PCR (qPCR)

The total RNA for each cell group was extracted and reverse-transcribed to cDNA in a total volume of 10 μl, in accordance with the manufacturer’s instructions (Cat. No. RR036A; TaKaRa, Tokyo, Japan). Fold inductions were calculated using the cycle threshold ΔΔCt method. qPCR was performed at 95 °C (30 s) followed by 40 cycles at 95 °C (5 s)/60 °C (30 s). SYBR green intercalating dye (Cat. No. RR820L; TaKaRa) was used for signal detection. For each sample, the number of cycles required to generate a given threshold signal (Ct) was recorded, and the results are shown as 2−ΔΔCt.

Sequences of the primers used in this study were as follows: GAPDH: 5′-GGAGCGAGATCCCTCCAAAAT-3′ (sense) and 5′-GGCTGTTGTCATACTTCTCATGG-3′ (antisense); TNF-α: 5′-CCTCTCTCTAATCAGCCCTCTG-3′ (sense) and 5′-GAGGACCTGGGAGTAGATGAG-3′ (antisense); VE-Cadherin: 5′-CGAGAGCTACACGTTCACGG-3′ (sense) and 5′-GGGTGTCGAGGGAAAAATAGG-3′ (antisense); Claudin-5: 5′-CTCTGCTGGTTCGCCAACAT-3′ (sense) and 5′-CAGCTCGTACTTCTGCGACA-3′ (antisense); Cx-43: 5′-GGTGACTGGAGCGCCTTAG-3′ (sense) and 5′-GCGCACATGAGAGATTGGGA-3′ (antisense).

### Western blot

For western blot analysis of TNF-α in supernatants (s-TNF-α), HPMEC supernatants were precipitated with one volume of methanol and one-quarter volume of chloroform. The precipitate was then washed in one volume of methanol and re-suspended in 60 μl of sodium dodecyl sulphate (SDS) loading buffer^39^. Cells were washed twice with PBS before protein extraction, and the collected cells were then lysed on ice for 30 min with radioimmunoprecipitation assay (RIPA) (Cat. No. P00013C, Beyotime Biotechnology, Co., Ltd., Beijing, China) lysis buffer containing an inhibitor cocktail (100:1) (Cat. No. HY-K0010; Medchemexpress, Monmouth Junction, NJ, USA) and phenylmethylsulfonyl fluoride (PMSF, 100:1) (Cat. No. ST506, Beyotime Biotechnology, Co., Ltd). The supernatant was then collected after centrifugation at 12,000 rpm for 15 min. The concentration of protein was estimated using a BCA Protein Quantification Kit (Cat. No. 23227; Thermo Fisher Scientific, Waltham, MA, USA), and loading buffer based on the concentration. After boiling at 100 °C for 10 min, a 30 µg protein sample was analysed by western blotting on 4–12% SDS-PAGE precast gels (Cat. No. NP0335, Invitrogen; Thermo Fisher Scientific). The 30 μl supernatant protein samples were separated by electrophoresis using 15% Tris-glycine polyacrylamide gels, then transferred to a polyvinylidene difluoride (PVDF) membrane (Cat. No. IPVH00010; Merck Millipore, Germany). Non-specific binding was blocked in Tris-buffered saline with 0.1% Tween-20 (TBST) containing 5% skimmed milk at room temperature for 1 h. The membranes were incubated with primary antibody overnight at 4 °C. Primary antibodies included VE-Cadherin (1:1000, #2500; Cell Signaling Technology), Claudin-5 (1:1000, ab15106; Abcam, Cambridge, MA, USA), Connexin-43 (1:8000, ab11370; Abcam), Akt (1:1000, #4685; Cell Signaling Technology), p-Akt (1:2000, #4060; Cell Signaling Technology), TNF-α (1:1000, #6945; Cell Signaling Technology), and GAPDH (1:1000, #5174; Cell Signaling Technology). The membranes were then washed three times for 10 min each in TBST. Next, the membranes were incubated with a horseradish peroxidase (HRP)-conjugated secondary antibody (1:5000, #7074; Cell Signaling Technology) for 2 h and washed three times for 10 min each in TBST. The protein bands were visualized by enhanced chemiluminescence kit (Cat. No. 70-P1421; MultiSciences Biotech, Co., Ltd., Hangzhou, China) and exposed to X-ray film.

### Immunofluorescence staining

Cells were washed three times for 5 min each with PBS, fixed for 15 min in 4% paraformaldehyde, washed three times in PBS, and treated for 5 min with 0.1% Triton X-100 to permeabilize the cell membranes. Next, 5% BSA was used to block cells for 1 h at room temperature. The cells were then incubated with primary antibody overnight at 4 °C using VE-Cadherin (1:200, #2500; Cell Signaling Technology), Claudin-5 (1:200, ab15106; Abcam), and Cx-43 antibodies (1:1000, ab11370; Abcam). After washing three times with PBS, the cells were incubated with Donkey Anti-Rabbit IgG H&L (Alexa Fluor 594) (1:500, ab150076; Abcam) or Goat Anti-Rabbit IgG H&L (Alexa Fluor 488) (1:500, ab150077; Abcam) for 1 h in the dark at room temperature. The cells were again washed three times with PBS and treated for 10 min with DAPI (1:2000, Cat. No. D9564; Sigma-Aldrich) to stain the cell nuclei. Finally, the cells were washed three times again in PBS and observed under a confocal microscope.

### Statistical analysis

All results are expressed as the mean ± standard deviation (SD). The data were analysed using two-tailed Student’s t-tests (for two-group comparisons) or one-way analysis of variance (ANOVA, for multiple-group comparisons) in GraphPad Prism 7.0 software (GraphPad Software Inc., San Diego, CA, USA). Values of P < 0.05 were considered to be statistically significant.

### Data Availability

The datasets generated during and/or analyzed during the current study are available from the corresponding author on reasonable request.

## Electronic supplementary material

Supplementary Information
