# Supplementary material for: Different concentrations of lipopolysaccharide regulate barrier function through the PI3K/Akt signalling pathway in human pulmonary microvascular endothelial cells
Source: Sci Rep. 2018 Jul 2;8:9963. doi: 10.1038/s41598-018-28089-3 (PMC6028427; doi:10.1038/s41598-018-28089-3)
Supplement: Supplementary file 1 — Supplementary Information [file 41598_2018_28089_MOESM1_ESM.pdf]

Different concentrations of lipopolysaccharide regulate barrier function through the PI3K/Akt signalling pathway in human pulmonary microvascular endothelial cells

Xia Zheng [1*], Wang Zhang[1*] & Xiaotong Hu[2]

[1]Department of Critical Care Medicine, The First Affiliated Hospital, College of Medicine, Zhejiang University, Hangzhou, Zhejiang, 310003, P.R. China.

[2]State Key Laboratory for Diagnosis and Treatment of Infectious Diseases, Collaborative Innovation Center for Diagnosis and Treatment of Infectious Diseases, The First Affiliated Hospital, College of Medicine, Zhejiang University, Hangzhou, Zhejiang, 310003, P.R. China.

[*]The authors contributed equally to this work

Correspondence and requests for materials should be addressed to X.Z. (email: zxicu@zju.edu.cn)

Supplementary Information

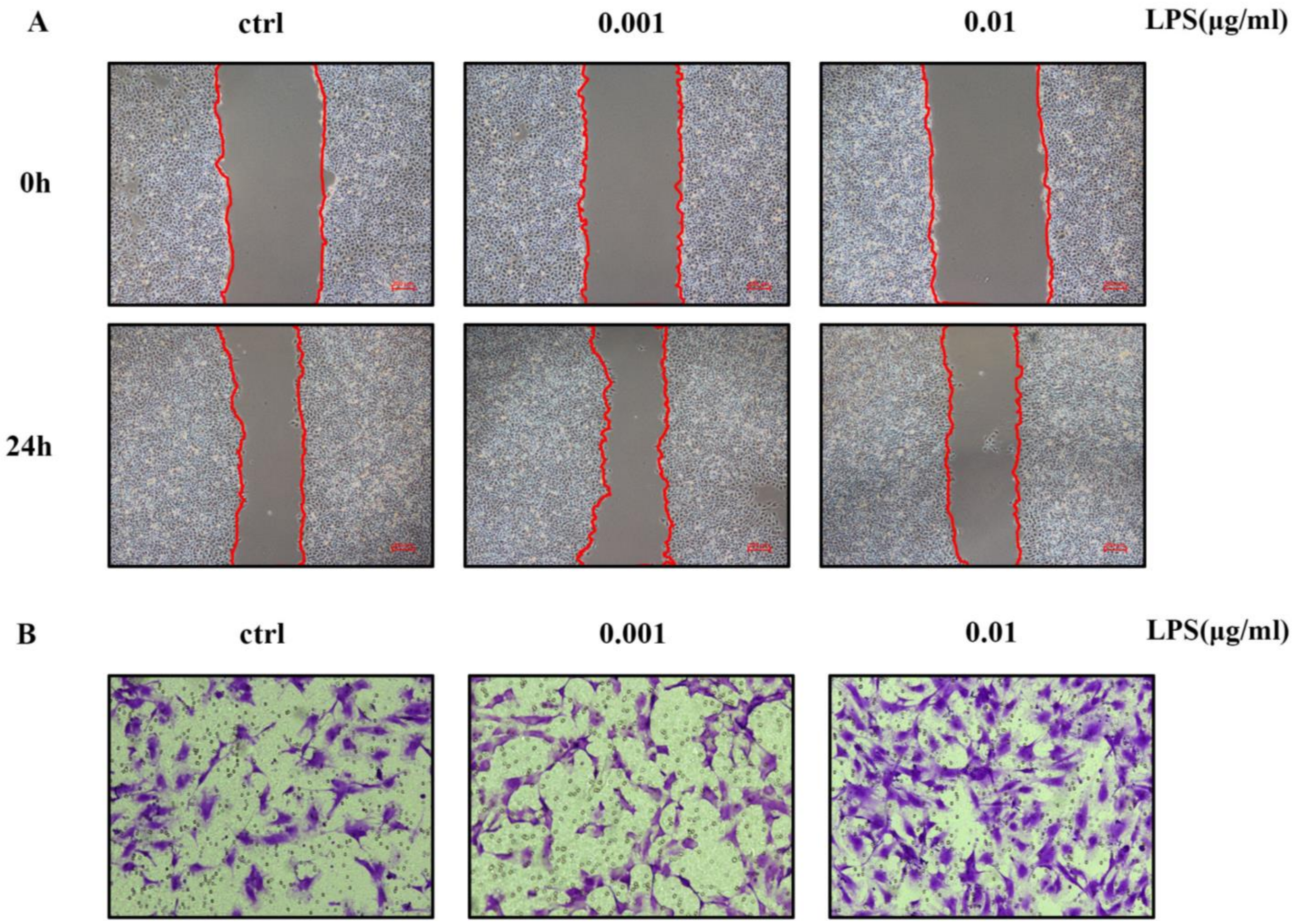

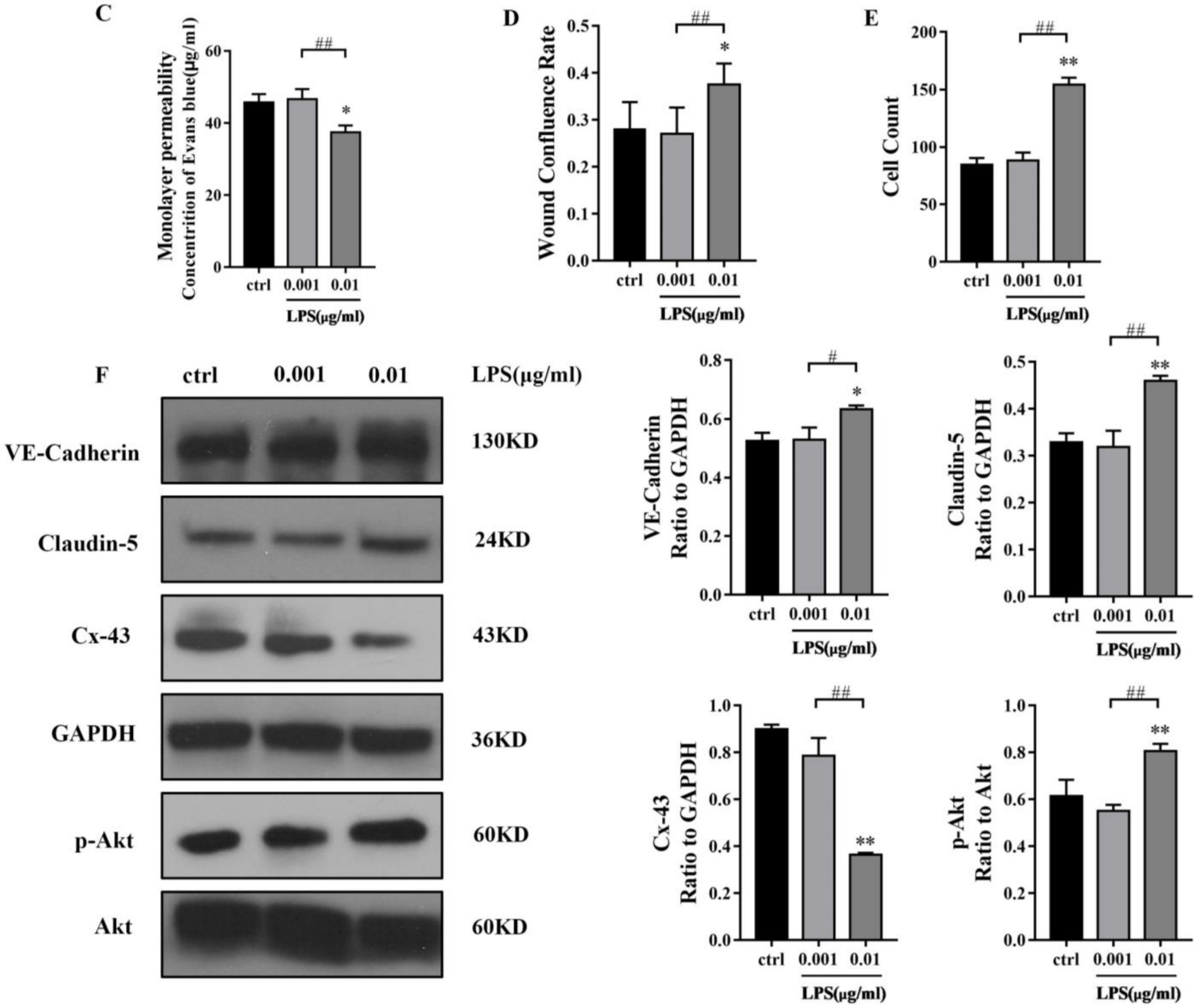

Supplementary Figure 1. The effects of LY294002 (50μM) on the migration, monolayer permeability, intercellular junction proteins expression and Akt phosphorylation in HPMECs. (**A, D)** The effects of LY294002 on scratch wound confluence in cultured HPMECs. (**A**) Representative images (**D**) Quantification of confluence rate at 24h (%wound confluence=(a-b) ×100% /a; a: Initial scratch wound area at 0h, b: Scratch wound area at 24h). (**B, E**) Cell transwell assay was used to measure migration. (**B**) Representative images. (**E**) Quantification of migrated cell number after 24h. (**C**) Monolayer permeability was measured by transwell- Evans blue (EB) assay. (**F**) Expression of intercellular junctions (VE-Cadherin, Cx-43, and Claudin-5), ratio of phosphorylated Akt to total Akt protein level were evaluated by western blot, relative protein levels were presented as a ratio of target protein to GAPDH. N.S. no significant difference vs control group. Error bars represent SD (standard deviation)

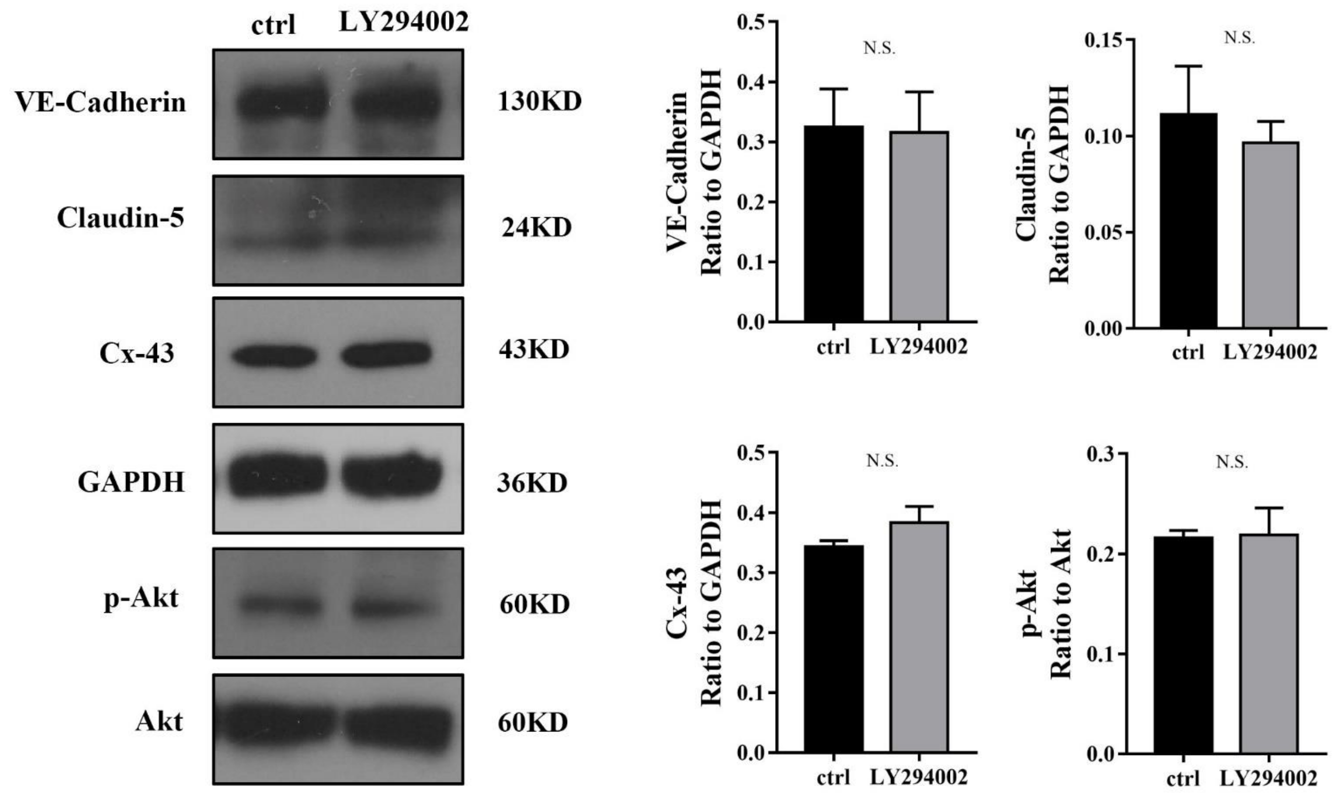

Supplementary Figure 2. The effects of 0.001 and 0.01μg/ml LPS on the migration, monolayer permeability, intercellular junction proteins expression and Akt phosphorylation in HPMECs. (**A, D)** Effects of LPS on scratch wound confluence in cultured HPMECs. (**A**) Representative images (**D**) Quantification of confluence rate at 24h. Compared with control group and 0.001μg/ml LPS group, the change of confluence rate after 0.01μg/ml treatment($P<0.05$) was significantly increased (**B, E**) Cell Transwell assay was used to measure migration. (**B**) Representative images. (**E**) Quantification of migrated cell number after 24h. Cell number in the 0.01μg/ml LPS group ($P<0.01$) was significantly higher than that of control group and 0.001μg/ml LPS group. (**C**) Monolayer permeability was measured by Transwell- EB assay. EB concentration in the lower chamber of 0.01μg/ml LPS group ($P<0.05$) was lower than in control group and 0.001μg/ml LPS group (**F**) Expression of intercellular junctions (VE-Cadherin, Cx-43, and Claudin-5), ratio of phosphorylated Akt to total Akt protein level were evaluated by western blot, relative protein levels were presented as a ratio of target protein to GAPDH. The levels of VE-Cadherin and Claudin-5 were remarkably up-regulated after 0.01μg/ml LPS treatment (VE-Cadherin: $P<0.05$, Claudin-5: $P<0.01$) than control group and 0.001μg/ml LPS group, the level of Cx-43 was down-regulated with 0.01ug/ml treatment($P<0.01$), ratio of phosphorylated Akt to total Akt protein level was increased in 0.01μg/ml LPS group ($P<0.01$). There were no significant differences in migration, monolayer permeability, intercellular junction proteins expression and Akt phosphorylation between 0.001μg/ml LPS group and control group. *$P<0.05$ vs. control group, **$P<0.01$ vs. control group, #$P<0.05$, ##$P<0.01$

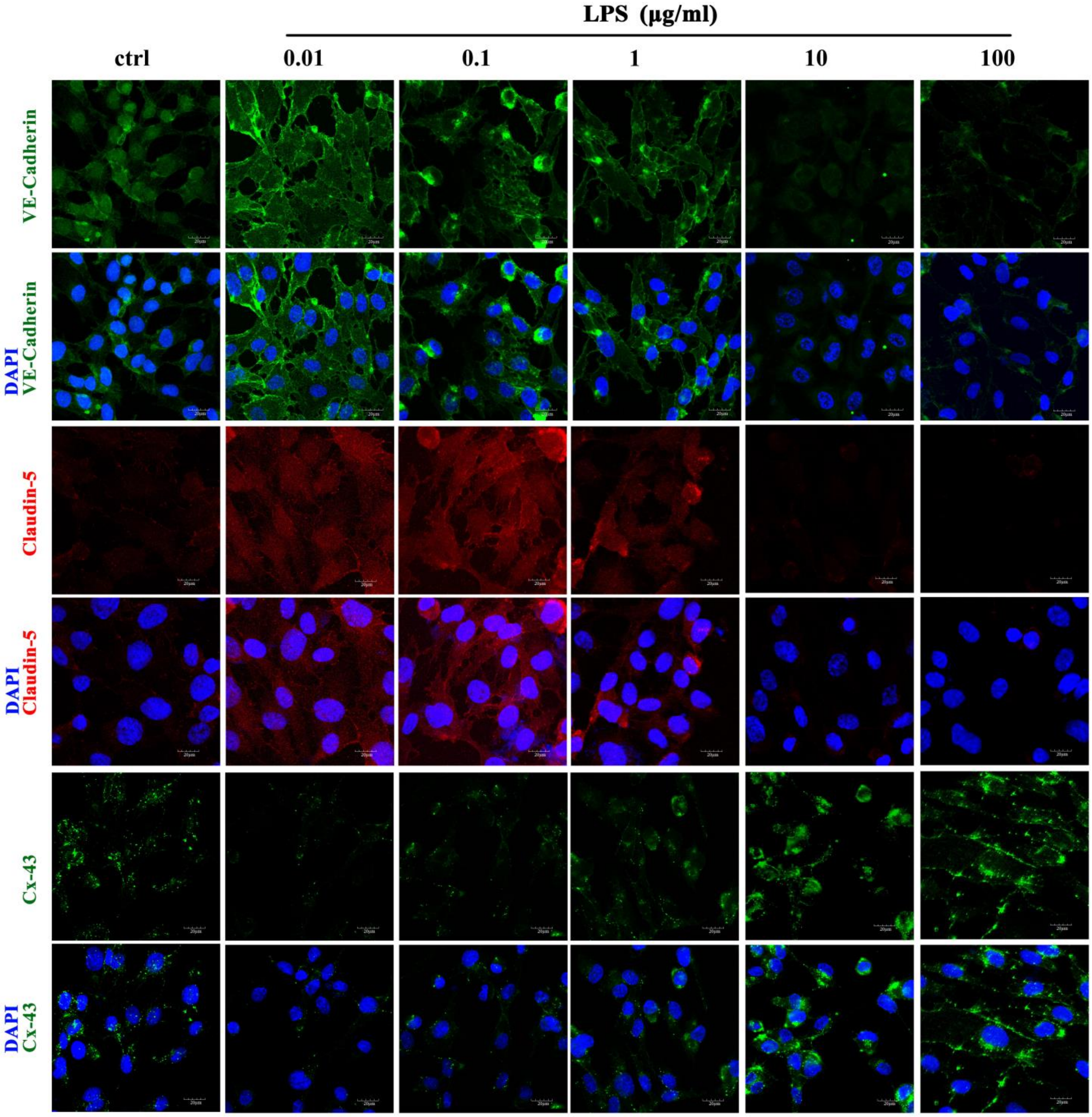

Supplementary Figure 4. Immunofluorescence staining of VE-Cadherin, Claudin-5, and Cx-43 in HPMECs treated with 0.01, 0.1, 1, 10 and 100 μg/ml LPS. Green indicates VE-Cadherin and Cx-43, while red indicates Claudin-5 and blue indicates nuclei. Scale bar: 20 μm

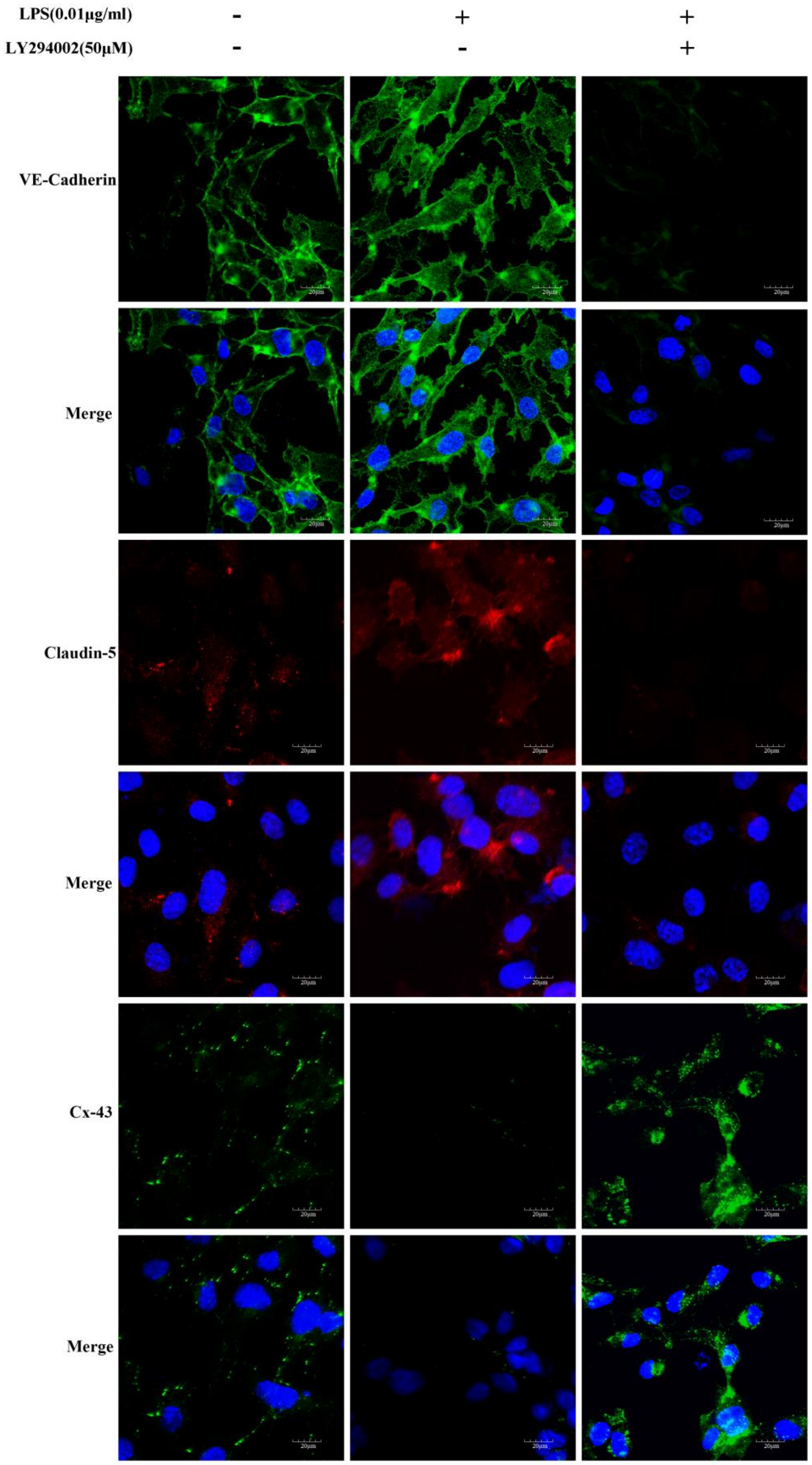

Supplementary-Figure 5. Immunofluorescence staining of VE-Cadherin, Claudin-5, and Cx-43 in HPMECs treated with 0.01 μg/ml LPS and 50 μM LY294002. Green indicates VE-Cadherin and Cx-43, while red indicates Claudin-5 and blue indicates nuclei. Scale bar: 20 μm
